# Vaccine-induced and naturally-acquired protection against Omicron and Delta symptomatic infection and severe COVID-19 outcomes, France, December 2021 to January 2022

**DOI:** 10.2807/1560-7917.ES.2022.27.16.2200250

**Published:** 2022-04-21

**Authors:** Milena Suarez Castillo, Hamid Khaoua, Noémie Courtejoie

**Affiliations:** 1 French national institute for statistical and economic studies (INSEE), Montrouge, France; 2 The Health and Solidarity Ministerial Statistical Department (DREES), Paris, France

**Keywords:** SARS-CoV-2, vaccination effectiveness, immunity, Test negative design

## Abstract

We assessed the protection conferred by naturally-acquired, vaccine-induced and hybrid immunity during the concomitant Omicron and Delta epidemic waves in France on symptomatic infection and severe COVID-19. The greatest levels of protection against both variants were provided by hybrid immunity. Protection against Omicron symptomatic infections was systematically lower and waned at higher speed than against Delta in those vaccinated. In contrast, there were little differences in variant-specific protection against severe inpatient outcomes in symptomatic individuals.

First designated by World Health Organization (WHO) as a variant of concern on 26 November 2021, the severe acute respiratory coronavirus 2 (SARS-Cov-2) Omicron variant (Phylogenetic Assignment of Named Global Outbreak (Pango) lineage designation: B.1.1.529) rapidly became dominant in multiple countries worldwide. By week 50 (13 to 19 December) 2021, this variant represented ca 10% of positive cases in France, while the Delta variant (Pango: B.1.617.2) was still largely circulating in the country [1].

We used large nationwide samples to investigate the impact of coronavirus disease (COVID-19) vaccination and/or previous SARS-CoV-2 infection on the risk of symptomatic infections, hospital admissions, intensive care unit (ICU) admissions and deaths attributable to the Omicron and Delta variants.

## Evidence of prior infections and vaccination history 

We analysed the matching at the individual level of three exhaustive nationwide French COVID-19 surveillance databases recording testing (SI-DEP), hospitalisations (SI-VIC), and vaccination (VAC-SI) from 13 December 2021 to 31 January 2022. We included reverse-transcription (RT)-PCR tests performed on individuals aged 18 years or over who reported symptoms in the 7 days before the time of screening. We defined a previous infection in an individual, as a confirmed SARS-CoV-2 infection that occurred at least 60 days before the time of testing (in coherence with the European Surveillance System definition of *suspected cases* of SARS-CoV-2 *reinfection*) [2], based on a previous positive RT-PCR, antigenic or serological test retrieved in the national surveillance database. Individuals without test history or with a test history indicating only negative results were classified as ‘without evidence of prior infection’. In France, subsets of positive RT-PCR samples are submitted to mutation screening, in order to characterise the likely variant. A set of predefined mutations are targeted by molecular screening to reliably identify the circulating variants, allowing to select cases of Omicron or Delta variants respectively (Supplementary materials, S2. Data description).

Over the 13 December 2021 to 31 January 2022 period, 3,332,529 individuals aged 18 years and over were tested by RT-PCR and reported symptoms at the time of screening. Of the latter, 87% (2,884,996/3,332,529) were successfully linked to vaccination data with non-missing data on comorbidities (described in Supplementary materials, S2. Data description). A total of 183,004 individuals were excluded because their vaccination status was atypical (e.g. people with a delayed second vaccine dose according to the schedule, or people having received at least one dose of Jansen vaccine within a two-dose vaccination cycle), leaving 2,701,992 included in the study. Of the latter, 80% (n = 2,164,491), had at least one test sampled before the study period, 9% of whom (n = 193,789) had a confirmed past infection history (Supplementary materials, Figure S-1.3). Among the 2,701,922 included individuals, the vast majority (69%, n = 1,875,935) had received two doses of Cominarty (BNT162b2, BioNTech-Pfizer, Mainz, Germany/New York, United States), 13% (n = 353,767) were not vaccinated, 9% (n = 233,254) had received two doses of Spikevax (mRNA-1273, Moderna, Cambridge, United States) and 3% (n = 89,812) two doses of Vaxzevria (ChAdOx1/nCoV-19, AstraZeneca, Cambridge, United Kingdom). The 6% (n = 149,154) remaining had either only received one dose of vaccine, or alternatively two doses, but each from a different vaccine product. In addition, 38% (n = 1,026,345) of the 2,701,992 included individuals had received a first booster dose; 74% with Cominarty (n = 762,429), 25% with Spikevax (n = 260,880), and under 1% (n = 3,036) with an undetermined vaccine product (which was inconsistent or missing in the records). Among the included individuals, 1,541,995 (57%) tested positive for SARS-CoV-2, with 761,744 (49%) classified as Omicron infection cases and 166,009 (11%) classified as Delta infection cases.

## Comparison of natural, vaccine-induced and hybrid immunity against symptomatic infections

First, we used a test-negative case–control design to estimate naturally-acquired, vaccine-induced or hybrid immunity (acquired from both vaccination and infection) against Delta- and/or Omicron symptomatic infections. The study population for the test-negative design analysis consisted of 926,376 Omicron- or Delta-positive cases, who could be matched, and 1,852,752 SARS-CoV-2-negative controls (two matched controls per case). Matching was based on age (10-year age brackets), sex (collected as a binary variable), residence (Nomenclature of territorial units for statistics (NUTS)-3 level), week of testing and presence of a comorbidity qualifying for prioritisation in the vaccination campaign according to the recommendations of the National Health Authority, as described previously [3].

## Vaccine-induced immunity against symptomatic infections

**Figure 1:**
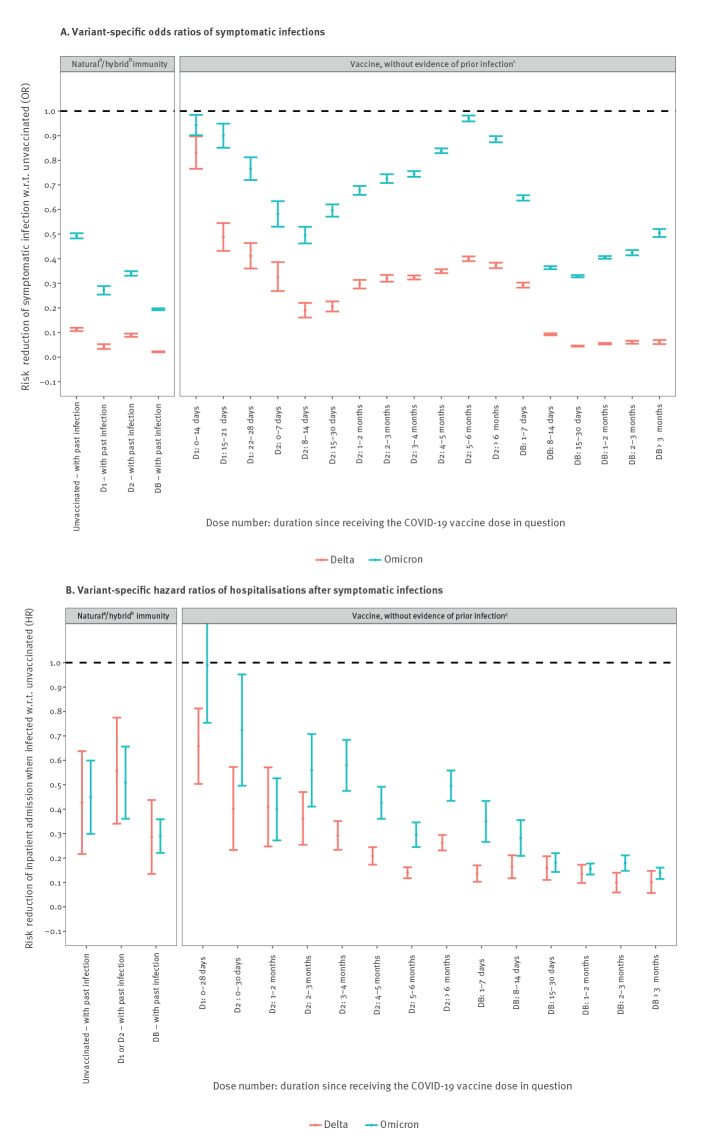
Variant-specific risk reduction of (A) symptomatic infections and (B) hospitalisations after symptomatic infections, among ≥ 18 year-olds, according to the time elapsed since each COVID-19 vaccine dose and evidence of prior SARS-CoV-2 infection, France, 13 December 2021–31 January 2022 (n = 761,744 Omicron and 166,009 Delta cases, respectively; n = 1,155,064 eligible controls)

COVID-19: coronavirus disease; D1: first vaccine dose; D2: second vaccine dose; DB: booster dose; HR: hazard ratio; OR: odds ratio; RT-PCR: reverse-transcription PCR; SARS-CoV-2: severe acute respiratory coronavirus 2; w.r.t: with reference to.

^a^ Natural immunity: individuals with evidence of prior SARS-CoV-2 infection; the causative variant for prior infection is unknown.

^b^ Hybrid immunity: individuals with evidence of prior SARS-CoV-2 infection and who received at least one dose of COVID-19 vaccine. The causative variant is unknown for prior infection.

^c^ Vaccine, without evidence of prior infection: individuals who received at least one COVID-19 vaccine dose and who were not linked to a prior SARS-CoV-2 positive test in the national surveillance database SI-DEP.

Among vaccinated persons aged 18 years and over without evidence of prior infection, the protection against Omicron symptomatic infections reached 43% in the first month following the second dose (Table 1, odds ratio (OR): 0.57; 95% confidence interval (CI): 0.55–0.59) and 64% 2 weeks after the first booster dose. These levels were largely below those reached against Delta symptomatic infections for similar immune statuses (respectively 78% and 91%). Additionally, the waning of protection after vaccine injections was much faster against Omicron than Delta infections (Figure 1A). Vaccine effectiveness against Omicron symptomatic infections decreased by 14 percentage points from 1 week after the first booster to 3 months after. By contrast, vaccine effectiveness against Delta symptomatic infections was stable above 90% up to 3 months following the first booster dose.

**Table 1:** Assessment among ≥ 18 year-olds of protection conferred by vaccination, natural immunity and hybrid immunity, against Omicron or Delta symptomatic infections and hospital admissions for COVID-19, France, 13 December 2021–31 January 2022 (n = 761,744 Omicron and 166,009 Delta cases, respectively; n = 1,155,064 eligible controls)

Immune status: time since named vaccine dose^b^	Omicron^a^			Delta^a^		
	Risk reduction^c^ against		Protection 1 − OR × HR	Risk reduction^c^ against		Protection 1 − OR × HR
	Symptomatic infection OR^d^ (95%CI)	Hospital admission among symptomatic cases HR^e^ (95%CI)	Protection(95%CI)	Symptomatic infection OR^d^ (95%CI)	Hospital admission among symptomatic cases HR^e^ (95%CI)	Protection (95%CI)
**Vaccinated (ref.: unvaccinated without prior infection evidence)**						
D1: 0 day–28 days	0.88 (0.86 to 0.91)	0.99 (0.75 to 1.23)	0.12 (−0.09 to 0.34)	0.62 (0.59 to 0.66)	0.66 (0.50 to 0.81)	0.59 (0.49 to 0.69)
D2: 0 days–30 days	0.57 (0.55 to 0.59)	0.72 (0.50 to 0.95)	0.59 (0.46 to 0.72)	0.22 (0.20 to 0.23)	0.40 (0.23 to 0.57)	0.91 (0.87 to 0.95)
D2: 1 month–2 months	0.68 (0.66 to 0.70)	0.40 (0.27 to 0.53)	0.73 (0.64 to 0.82)	0.30 (0.28 to 0.31)	0.41 (0.25 to 0.57)	0.88 (0.83 to 0.93)
D2: 2 months–3 months	0.73 (0.71 to 0.74)	0.56 (0.41 to 0.71)	0.59 (0.49 to 0.70)	0.32 (0.31 to 0.33)	0.36 (0.25 to 0.47)	0.88 (0.85 to 0.92)
D2: 3 months–4 months	0.74 (0.73 to 0.76)	0.58 (0.48 to 0.68)	0.57 (0.49 to 0.65)	0.32 (0.32 to 0.33)	0.29 (0.23 to 0.35)	0.91 (0.89 to 0.92)
D2: 4 months–5 months	0.84 (0.83 to 0.85)	0.43 (0.36 to 0.49)	0.64 (0.59 to 0.70)	0.35 (0.34 to 0.36)	0.21 (0.17 to 0.24)	0.93 (0.91 to 0.94)
D2: 5 months–6 months	0.97 (0.96 to 0.98)	0.30 (0.24 to 0.35)	0.71 (0.66 to 0.76)	0.40 (0.39 to 0.41)	0.14 (0.12 to 0.16)	0.94 (0.94 to 0.95)
D2: > 6 months	0.89 (0.87 to 0.90)	0.50 (0.43 to 0.56)	0.56 (0.51 to 0.62)	0.37 (0.36 to 0.38)	0.26 (0.23 to 0.29)	0.90 (0.89 to 0.91)
DB: 1 day –7 days	0.65 (0.64 to 0.66)	0.35 (0.27 to 0.43)	0.77 (0.72 to 0.83)	0.29 (0.28 to 0.30)	0.14 (0.10 to 0.17)	0.96 (0.95 to 0.97)
DB: 8 days–14 days	0.36 (0.36 to 0.37)	0.28 (0.21 to 0.36)	0.90 (0.87 to 0.92)	0.09 (0.09 to 0.10)	0.16 (0.12 to 0.21)	0.98 (0.98 to 0.99)
DB: 15 days–30 days	0.33 (0.32 to 0.33)	0.18 (0.14 to 0.22)	0.94 (0.93 to 0.95)	0.04 (0.04 to 0.05)	0.16 (0.11 to 0.21)	0.99 (0.99 to 1.00)
DB: 1 month–2 months	0.41 (0.40 to 0.41)	0.16 (0.13 to 0.18)	0.94 (0.93 to 0.95)	0.05 (0.05 to 0.06)	0.14 (0.10 to 0.17)	0.99 (0.99 to 0.99)
DB: 2 months –3 months	0.42 (0.41 to 0.43)	0.18 (0.15 to 0.21)	0.92 (0.91 to 0.94)	0.06 (0.05 to 0.07)	0.10 (0.06 to 0.14)	0.99 (0.99 to 1.00)
DB > 3 months	0.50 (0.49 to 0.52)	0.14 (0.11 to 0.16)	0.93 (0.92 to 0.94)	0.06 (0.05 to 0.07)	0.10 (0.06 to 0.15)	0.99 (0.99 to 1.00)
**Naturally-acquired and hybrid immunity** ^f^ **(ref.: unvaccinated without prior infection evidence)**						
Unvaccinated: NA	0.49 (0.48 to 0.50)	0.45 (0.30 to 0.60)	0.78 (0.70 to 0.85)	0.11 (0.11 to 0.12)	0.43(0.22 to 0.64)	0.95(0.93 to 0.98)
D1 or D2: NA	0.33 (0.32 to 0.34)	0.51 (0.36 to 0.66)	0.83 (0.78 to 0.88)	0.08 (0.08 to 0.09)	0.56 (0.34 to 0.77)	0.95 (0.94 to 0.97)
DB: NA	0.19 (0.19 to 0.20)	0.29 (0.22 to 0.36)	0.94 (0.93 to 0.96)	0.02 (0.02 to 0.02)	0.29 (0.13 to 0.44)	0.99 (0.99 to 1.00)

CI: confidence interval; COVID-19: coronavirus disease; D1: first vaccine dose; D2: second vaccine dose; DB: booster dose; HR: hazard ratio; NA: not applicable; OR: odds ratio; ref.: reference; RT-PCR: reverse-transcription PCR; SARS-CoV-2: severe acute respiratory coronavirus 2.

^a^ Delta (respective Omicron): laboratory-confirmed (RT-PCR) SARS-CoV-2 infection with mutation screening indicative of Delta (respective Omicron) variant [14].

^b^ Duration since receiving the COVID-19 vaccine dose in question, at presentation to the screening centre.

^c^ Risk reductions are relative to symptoms attributable respectively to the Delta or the Omicron variant.

^d^ Odds ratios of symptomatic infections, according to the time elapsed since each COVID-19 vaccine dose reception or according to evidence of prior infection.

^e^ Hazard ratios of hospitalisations after symptomatic infections, according to the time elapsed since each COVID-19 vaccine dose reception or according to evidence of prior infection.

^f^ Naturally-acquired immunity: individuals with evidence of prior SARS-CoV-2 infection; the causative variant for prior infection is unknown.

Delta (respective Omicron): laboratory-confirmed (RT-PCR) SARS-CoV-2 infection with mutation screening indicative of Delta (respective Omicron) variant [14].

Risk reductions are relative to symptoms attributable respectively to the Delta or the Omicron variant. The causative variant is unknown for prior infection.

Categories with few observations were joined when studying hospitalisations.

Each value in the graph is indicated by a dot. The whiskers around each dot are the 95% confidence intervals.

## Naturally-acquired and hybrid immunity against symptomatic infections

**Figure 2:**
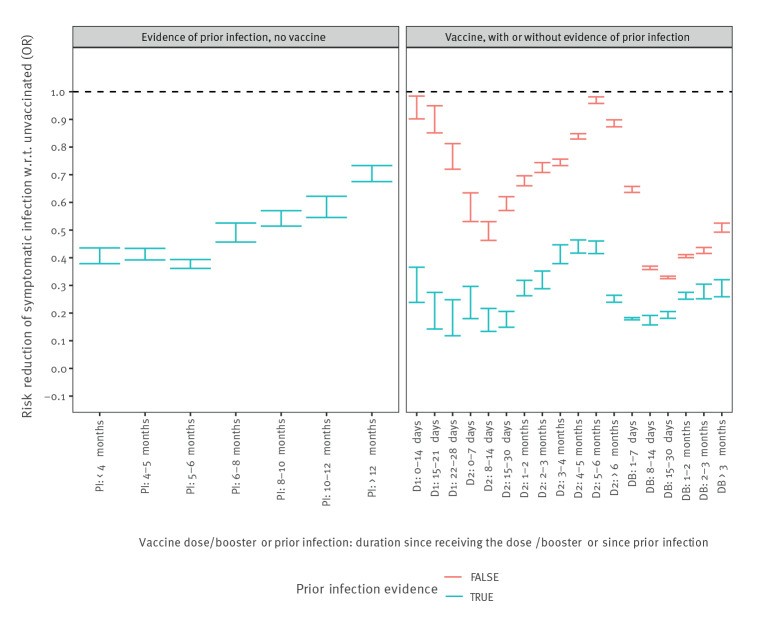
Odd ratios of Omicron-attributable symptomatic infections among ≥18 year-olds, according to the time elapsed since each COVID-19 vaccine dose and/or evidence of prior infection, France, 13 December 2021–31 January 2022 (n = 761,744 Omicron cases; n = 1,155,064 eligible controls)

COVID-19: coronavirus disease; D1: first vaccine dose; D2: second vaccine dose; DB: booster dose; OR: odds ratio; PI: prior infection; RT-PCR: reverse-transcription PCR; SARS-CoV-2: severe acute respiratory coronavirus 2; w.r.t: with reference to.

The protection conferred by a prior infection among unvaccinated persons was 51% against symptomatic infections with the Omicron variant (Table 1), while it was 89% with the Delta variant. Hybrid immunity (prior infection and at least one vaccine dose) reached 67% protection and 81% with a booster dose against symptomatic infection with the Omicron variant, and even higher levels (> 90%) were reached against Delta. Hybrid immunity against Omicron symptomatic infections could be studied in more details given the higher number of observations. We observed different levels of protection, but similar dynamics given the time since the last injection, in individuals without or with evidence of prior infection (Figure 2). For the latter however, the highest levels of protection were obtained in the first month following the receipt of (any) COVID-19 vaccine dose. In unvaccinated individuals, the decrease in the protection against Omicron symptomatic infections with duration since prior infection may be interpreted as waning immunity or differential protection from different strains.

Omicron: laboratory-confirmed (RT-PCR) SARS-CoV-2 infection with mutation screening indicative of Omicron variant [14].

Each value in the graph is indicated by a dot. The whiskers around each dot are the 95% confidence intervals.

## Protection against severe outcomes by immune status

We performed a survival analysis among COVID-19-confirmed individuals with symptoms, to estimate a possible additional risk reduction against severe forms of the disease, provided by natural and/or vaccine-induced immunity. The study population consisted of the 927,753 persons with confirmed SARS-CoV-2 symptomatic infections associated with either the Omicron or the Delta variant. Among the 761,744 Omicron cases, there were 2,994 hospitalisations, 387 ICU admissions and 407 inpatient deaths linked in SI-VIC. Among the 166,009 Delta cases, there were 3,367 hospitalisations, 1,006 ICU admissions and 524 inpatient deaths recorded in SI-VIC.

Upon a symptomatic infection with either the Omicron or the Delta variant, most prior-immunity (naturally-acquired, vaccine-induced or hybrid) conferred protection levels against disease progression leading to hospital admission independent of the variant (Figure 1). Nevertheless, from 3 months after reception of the second dose to the week following the booster dose, the protection against severe outcomes due to infections with Omicron was significantly lower than that due those with the Delta variant. These conclusions are similar for protection against ICU-admissions and in-hospital deaths (Table 2).

**Table 2:** Risk Reduction (hazard ratio) against hospital admission, ICU admission and inpatient death, among symptomatic cases aged ≥ 18 years, according to the time elapsed since each COVID-19 vaccine dose and evidence of prior infection, France, 13 December 2021–31 January 2022 (n = 761,744 Omicron and 166,009 Delta cases, respectively)

Immune status: time since named vaccine dose^b^	Omicron^a^			Delta^a^		
	Hospital admission HR^c^ (95%CI)	ICU admission HR^c^ (95%CI)	Death HR^c^ (95%CI)	Hospital admission HR^c^ (95%CI)	ICU admission HR^c^ (95%CI)	Death HR^c^ (95%CI)
**Vaccinated (ref.: unvaccinated without prior infection evidence)**						
D1: 0–28 days	0.99 (0.75 to 1.23)	1.09 (0.49 to 1.69)	1.09 (0.53 to 1.65)	0.66 (0.50 to 0.81)	0.43 (0.21 to 0.65)	0.93 (0.48 to 1.37)
D2: 0–30 days	0.72 (0.50 to 0.95)	0.54 (0.06 to 1.02)	0.71 (0.14 to 1.29)	0.40 (0.23 to 0.57)	0.32 (0.04 to 0.60)	0.44 (0.01 to 0.87)
D2: 1–2 months	0.40 (0.27 to 0.53)	0.32 (0.06 to 0.59)	0.38 (0.10 to 0.67)	0.41 (0.25 to 0.57)	0.52 (0.21 to 0.84)	0.14 (−0.13 to 0.42)
D2: 2–3 months	0.56 (0.41 to 0.71)	0.22 (0.00 to 0.43)	0.12 (−0.05 to 0.29)	0.36 (0.25 to 0.47)	0.35 (0.16 to 0.54)	0.11 (−0.04 to 0.26)
D2: 3–4 months	0.58 (0.48 to 0.68)	0.25 (0.09 to 0.42)	0.43 (0.22 to 0.65)	0.29 (0.23 to 0.35)	0.18 (0.10 to 0.26)	0.31 (0.12 to 0.49)
D2: 4–5 months	0.43 (0.36 to 0.49)	0.15 (0.07 to 0.24)	0.30 (0.14 to 0.45)	0.21 (0.17 to 0.24)	0.17 (0.12 to 0.23)	0.37 (0.20 to 0.53)
D2: 5–6 months	0.30 (0.24 to 0.35)	0.19 (0.11 to 0.28)	0.32 (0.15 to 0.48)	0.14 (0.12 to 0.16)	0.10 (0.07 to 0.13)	0.20 (0.11 to 0.28)
D2: > 6 months	0.50 (0.43 to 0.56)	0.32 (0.21 to 0.42)	0.51 (0.36 to 0.65)	0.26 (0.23 to 0.29)	0.14 (0.11 to 0.18)	0.35 (0.25 to 0.44)
DB: 1–7 days	0.35 (0.27 to 0.43)	0.12 (0.02 to 0.22)	0.29 (0.07 to 0.50)	0.14 (0.10 to 0.17)	0.06 (0.03 to 0.10)	0.29 (0.15 to 0.43)
DB: 8–14 days	0.28 (0.21 to 0.36)	0.12 (0.02 to 0.21)	0.14 (0.00 to 0.28)	0.16 (0.12 to 0.21)	0.07 (0.02 to 0.12)	0.24 (0.09 to 0.39)
DB: 15–30 days	0.18 (0.14 to 0.22)	0.13 (0.07 to 0.20)	0.18 (0.08 to 0.28)	0.16 (0.11 to 0.21)	0.15 (0.07 to 0.23)	0.15 (0.02 to 0.29)
DB: 1–2 months	0.16 (0.13 to 0.18)	0.06 (0.03 to 0.08)	0.15 (0.10 to 0.21)	0.14 (0.10 to 0.17)	0.13 (0.07 to 0.19)	0.16 (0.06 to 0.25)
DB: 2–3 months	0.18 (0.15 to 0.21)	0.08 (0.04 to 0.13)	0.14 (0.08 to 0.20)	0.10 (0.06 to 0.14)	0.08 (0.00 to 0.15)	0.09 (0.01 to 0.16)
DB > 3 months	0.14 (0.11 to 0.16)	0.05 (0.01 to 0.09)	0.13 (0.08 to 0.17)	0.10 (0.06 to 0.15)	0.03 (−0.03 to 0.09)	0.10 (0.01 to 0.19)
**Naturally-acquired or hybrid immunity** ^d^ **(ref.: unvaccinated without prior infection evidence)**						
Unvaccinated: NA	0.45 (0.30 to 0.60)	0.14 (−0.05 to 0.33)	0.24 (−0.09 to 0.58)	0.43 (0.22 to 0.64)	0.54 (0.10 to 0.97)	1.06 (0.02 to 2.10)
D1 or D2: NA	0.51 (0.36 to 0.66)	0.42 (0.12 to 0.72)	0.34 (0.07 to 0.61)	0.56 (0.34 to 0.77)	0.39 (0.08 to 0.71)	0.90 (0.17 to 1.62)
DB: NA	0.29 (0.22 to 0.36)	0.16 (0.05 to 0.28)	0.19 (0.06 to 0.32)	0.29 (0.13 to 0.44)	0.13 (−0.05 to 0.30)	0.11 (−0.11 to 0.33)

CI: confidence interval; COVID-19: coronavirus disease; D1: first vaccine dose; D2: second vaccine dose; DB: booster dose; HR: hazard ratio; NA: not applicable; ref.: reference; RT-PCR: reverse-transcription PCR; SARS-CoV-2: severe acute respiratory coronavirus 2.

^a^ Delta (respective Omicron): laboratory-confirmed (RT-PCR) SARS-CoV-2 infection with mutation screening indicative of Delta (respective Omicron) variant [14].

^b^ Duration since receiving the COVID-19 vaccine dose in question, at presentation to the screening centre.

^c^ Hazard ratios of hospitalisations after symptomatic infections, according to the time elapsed since each COVID-19 vaccine dose reception or according to evidence of prior infection.

^d^ Naturally-acquired immunity: individuals with evidence of prior SARS-CoV-2 infection; the causative variant for prior infection is unknown.

Risk reductions are relative to symptomatic COVID-19 attributable respectively to the Delta or the Omicron variant. The causative variant is unknown for prior infection.

## Discussion

Before the Omicron variant upsurge, observational studies indicated that naturally-acquired immunity offered equal or greater protection against SARS-CoV-2 infections, than receiving two doses of an mRNA vaccine [4]. Nonetheless, early evidence pointed towards a reduced immunity against Omicron infections following both vaccination [5,6] and infection [7]. Prior studies [5,8] estimated vaccine effectiveness against symptomatic Omicron or Delta infection according to the time elapsed since the second and booster doses, in the context of vaccines used in England (Cominarty/Vaxzevria) [5] or of the SpikeVax vaccine in California [8]. Our findings are similar when considering the vaccine courses that are predominant in the French context (Cominarty/Spikevax vaccine primary course and Cominarty/Spikevax first booster). As in the English study, we also found an additional risk reduction (hazard ratio) against hospital admission following Omicron or Delta infection in those vaccinated [6]. With a research design similar to the current study in Qatar [7], an effectiveness of previous infection in preventing reinfection of 92.0% (95%CI: 87.9 to 94.7) was estimated against the Delta variant, and of 56.0% (95%CI:  50.6 to 60.9) against the Omicron variant. With respect to severe, critical, or fatal Covid-19, the effectiveness was estimated to be 100% (95%CI: 43.3 to 100) against the Delta variant, and 87.8% (95%CI:  47.5 to 97.1) against the Omicron variant [7]. These estimates are not statistically different from their respective findings in the current study. Moreover, our results go into further details, considering both the time elapsed since infection and a potential interaction with vaccine-induced immunity. In our study, the first booster is associated with a lower risk of COVID-19 both among those with a primary vaccination course as well as those with natural immunity. This is consistent with other results obtained during an Omicron variant surge [9]. Against the Omicron variant, our findings are in favour of a greater or equal protection of natural immunity in the first six months following prior infection compared to a vaccine primary course, but of a lower protection compared to a vaccine primary course followed by a recent booster dose. Our work also suggests that hybrid immunity combining a recent booster dose and a past infection confers the highest level of protection.

The exhaustiveness of the databases used is the great strength of this study. We used a test-negative design to reduce selection biases that are difficult to measure such as health-seeking behaviour, access to testing and case ascertainment. 

Nevertheless, there are some limitations to the study. Test-negative designs rely on strong assumptions [10,11]. The definition of immune statuses was error prone as many infections remain undetected, given the frequency of asymptomatic infections, and imperfections in data linkage that may impair the tracing of past infections (Supplementary materials, S2). The level of protection conferred by hybrid immunity may thus be subject to downward bias. Most of the prior infections considered in our sample are attributable to other variants than Omicron, given the delay of 60 days used to ascertain a reinfection case. Thus, this study does not inform on the natural or hybrid immunity attributable to Omicron infection. In our study period, prior infections are likely homogeneous with respect to symptomatology and thus ascertainment, which will not necessarily be the case in the future due to the potentially milder symptomatology of the Omicron variant [6,9,12,13]. To maximise external validity, we used a rather liberal definition of the Delta and Omicron variants based on screening methods. Nonetheless, a more conservative definition as in Auvigne et al. (2022) would have little impact on our results (Supplementary materials) [13].

## Conclusion

Our findings indicate a greater ability of the Omicron variant to escape natural and vaccine-induced protection, combined with a faster decline in vaccine protection for this variant compared to the Delta variant. Importantly, we observe a fast decline in protection against Omicron symptomatic infection following a first booster, a decline that is not observed against disease progression to severe forms of COVID-19.

## Supplementary Data

1462000 Supplementary Materials
