# Author's correction for Euro Surveill. 2022;27(16)

**DOI:** 10.2807/1560-7917.ES.2022.27.17.220428c

**Published:** 2022-04-28

**Authors:** Eurosurveillance editorial team

**Affiliations:** 1 European Centre for Disease Prevention and Control (ECDC), Stockholm, Sweden

In the article ‘Vaccine-induced and naturally-acquired protection against Omicron and Delta symptomatic infection and severe COVID-19 outcomes, France, December 2021 to January 2022’ by Suarez Castillo et al., published on 21 April 2022, the number ‘2,701,992’ was mistyped as ‘2,701,922’ in one sentence. This was corrected on 22 April 2022 at the request of the authors.

