# Supplementary material for: Vaccine-induced and naturally-acquired protection against Omicron and Delta symptomatic infection and severe COVID-19 outcomes, France, December 2021 to January 2022
Source: Euro Surveill. 2022 Apr 21;27(16):2200250. doi: 10.2807/1560-7917.ES.2022.27.16.2200250 (PMC9027152; doi:10.2807/1560-7917.ES.2022.27.16.2200250)
Supplement: Supplementary Materials [file 2200250_SupplementaryMaterials.pdf]

Supplementary materials

This supplementary material is hosted by *Eurosurveillance* as supporting information alongside the article `Vaccine-induced` and naturally-acquired protection against Omicron and Delta symptomatic infection and severe COVID-19 outcomes, France, December 2021 to January 2022 on behalf of the authors who remain responsible for the accuracy and appropriateness of the content. The same standards for ethics, copyright, attributions and permissions as for the article apply. Supplements are not edited by Eurosurveillance and the journal is not responsible for the maintenance of any links or email addresses provided therein.

S 1. Study Population

Figure S-1.1 • Daily counts of controls, symptomatic and hospitalized cases (averaged over the last 7 days), data collected from December 13, 2021 to January 31, 2022

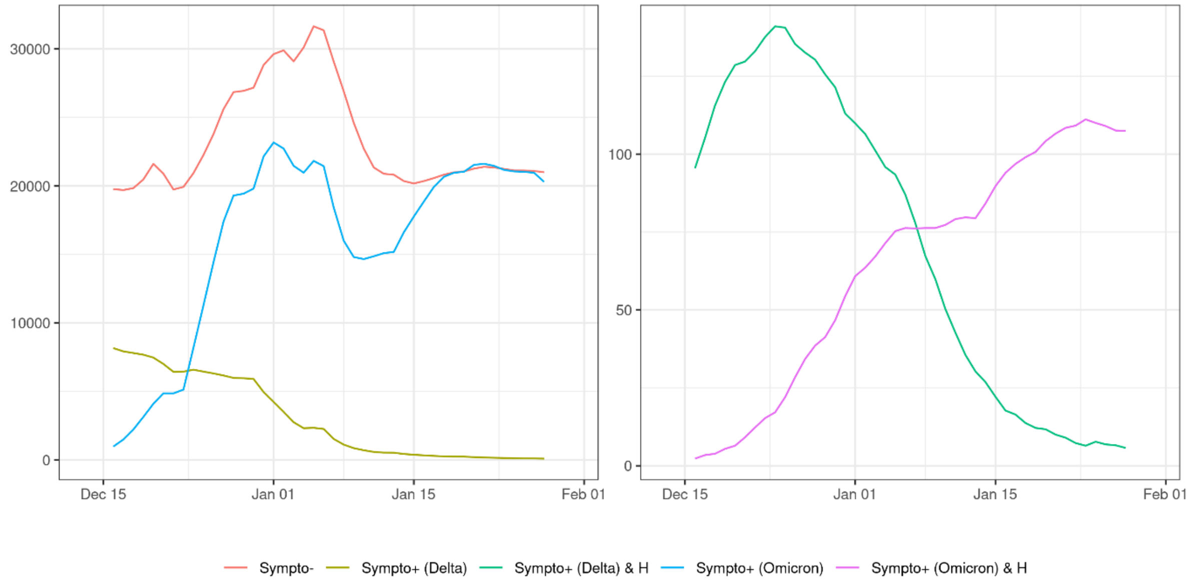

Abbreviations: Sympto+ (cases): symptomatic individuals with a laboratory confirmed SARS-CoV-2 infection (cases); Sympto+ & H (hospitalized cases): symptomatic individuals with a laboratory confirmed SARS-CoV-2 infection and hospital admission for Covid-19. Sympto- (controls): individuals with symptoms non-related to SARS-CoV-2 infection.

Figure S-2.2 • Proportion of controls and cases with evidence of prior SARS-Cov-2 infection, data collected from December 13, 2021 to January 31, 2022

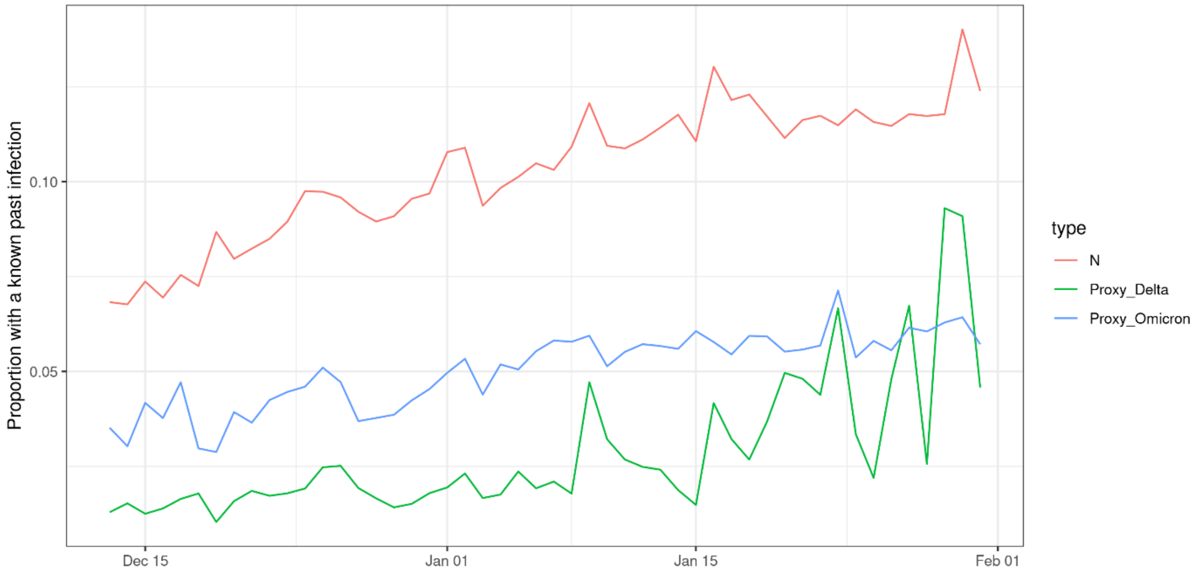

Abbreviations: Proxy_Delta (Delta cases): symptomatic individuals with a laboratory confirmed SARS-CoV-2 infection with screened mutations indicative of the Delta variant; Proxy_Omicron (Omicron cases): symptomatic individuals with a laboratory confirmed SARS-CoV-2 infection with screened mutations indicative of the Omicron variant;. N (controls): individuals with symptoms non-related to SARS-CoV-2 infection.

Figure S-1.3 • Counts of cases and controls with prior evidence of infection by date of past infection detection, data collected from December 13, 2021 to January 31, 2022

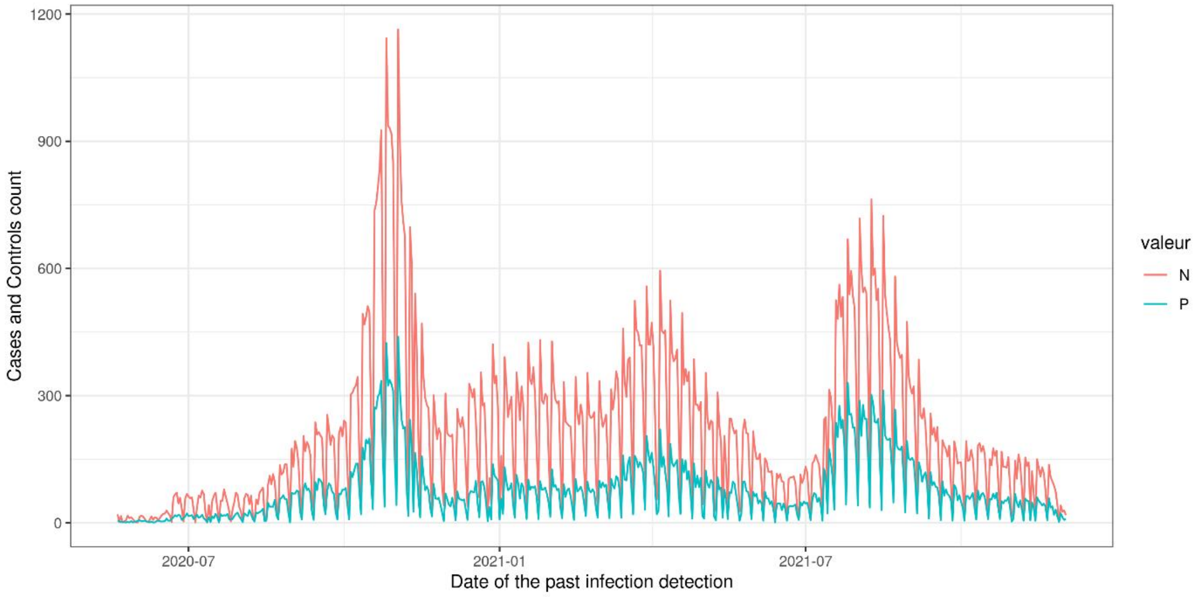

Abbreviations: P (cases): symptomatic individuals with a laboratory confirmed SARS-CoV-2 infection (cases) with screened mutations indicative of the Delta or Omicron variant; N (controls): individuals with symptoms non-related to SARS-CoV-2 infection.

Table S-1.1 • Supporting information for crude odd-ratios in the assessment among ≥ 18 year-olds of protection conferred by vaccination, natural immunity and hybrid immunity, against Omicron or Delta symptomatic infections, France, 13 December 2021–31 January 2022 (n = 761,744 Omicron and 166,009 Delta cases, respectively; n = 1,155,064 eligible controls)

Immune status: time since named vaccine dose	Omicron analysis			Delta analysis		
	Symptomatic cases with Omicron	Symptomatic controls negative to SARS-Cov-2 matched with cases	Crude OR	Symptomatic cases with Delta	Symptomatic controls negative to SARS-Cov-2 matched with cases	Crude OR
**Vaccinated (ref.: unvaccinated without prior infection evidence)**						
Ref.	93217	130594	1	47892	40014	1
D1: 0 day –	10882	20900	0.73	3306	5873	0.47
D2: 0 days–30 days	5404	12531	0.6	978	3097	0.26
D2: 1 month–2 months	9617	18092	0.74	1799	4639	0.32
D2: 2 months–3 months	11675	21442	0.76	3613	8915	0.34
D2: 3 months–4 months	36966	68677	0.75	14471	36258	0.33
D2: 4 months–5 months	118035	193258	0.86	26553	53280	0.42
D2: 5 months–6 months	132289	172715	1.07	33020	48073	0.57
D2: > 6 months	75863	96757	1.1	14529	20166	0.6
DB: 1 day –7 days	33335	62262	0.75	7680	14649	0.44
DB: 8 days–14 days	28334	91398	0.43	3501	19163	0.15
DB: 15 days–30 days	59600	204273	0.41	2748	29257	0.08
DB: 1 month–2 months	86467	219736	0.55	1947	14217	0.11
DB: 2 months –3 months	11628	26475	0.62	557	3278	0.14
DB > 3 months	7745	14792	0.73	312	1595	0.16

**Naturally-acquired and hybrid immunity (ref.: unvaccinated without prior infection evidence)**						
Unvaccinated: NA	14292	40616	0.49	1332	9860	0.11
D1 or D2: NA	9084	33508	0.38	984	7239	0.11
DB: NA	16209	93258	0.24	512	11895	0.04

Table S-1.2 • Supporting information for crude Hazard-ratio in the assessment among ≥ 18 year-olds of protection conferred by vaccination, natural immunity and hybrid immunity, against hospital admissions for COVID-19 among cases with Omicron or Delta symptomatic infections, France, 13 December 2021–31 January 2022 (n = 761,744 Omicron and 166,009 Delta cases, respectively; n = 1,155,064 eligible controls)

**Immune status: time since named vaccine dose**	**Omicron**			**Delta**		
	**Hospital admission / Symptomatic cases (Crude Rate Ratio)**	**ICU admission / Symptomatic cases (Crude Rate Ratio)**	**Inpatient death / Symptomatic cases (Crude Rate Ratio)**	**Hospital admission / Symptomatic cases (Crude Rate Ratio)**	**ICU admission / Symptomatic cases (Crude Rate Ratio)**	**Inpatient death / Symptomatic cases (Crude Rate Ratio)**
**Vaccinated (ref.: unvaccinated without prior infection evidence)**						
Ref.	847/93217(1)	152/93217(1)	146/93217(1)	2107/47892(1)	698/47892(1)	315/47892(1)
D1: 0–	106/10882(1.07)	18/10882(1.01)	19/10882(1.11)	104/3306(0.72)	18/3306(0.37)	27/3306(1.24)
D2: 0–30 days	41/5404(0.83)	5/5404(0.57)	6/5404(0.71)	22/978(0.51)	5/978(0.35)	4/978(0.62)
D2: 1–2 months	40/9617(0.46)	6/9617(0.38)	7/9617(0.46)	25/1799(0.32)	11/1799(0.42)	1/1799(0.08)
D2: 2–3 months	59/11675(0.56)	4/11675(0.21)	2/11675(0.11)	45/3613(0.28)	13/3613(0.25)	2/3613(0.08)
D2: 3–4 months	141/36966(0.42)	10/36966(0.17)	16/36966(0.28)	101/14471(0.16)	21/14471(0.1)	11/14471(0.12)
D2: 4–5 months	211/118035(0.2)	15/118035(0.08)	14/118035(0.08)	144/26553(0.12)	45/26553(0.12)	20/26553(0.11)
D2: 5–6 months	171/132289(0.14)	25/132289(0.12)	15/132289(0.07)	174/33020(0.12)	50/33020(0.1)	19/33020(0.09)

D2: > 6 months	390/75863(0.57)	56/75863(0.45)	56/75863(0.47)	332/14529(0.52)	70/14529(0.33)	61/14529(0.64)
DB: 1–7 days	75/33335(0.25)	6/33335(0.11)	7/33335(0.13)	68/7680(0.2)	13/7680(0.12)	17/7680(0.34)
DB: 8–14 days	63/28334(0.24)	6/28334(0.13)	4/28334(0.09)	49/3501(0.32)	9/3501(0.18)	10/3501(0.43)
DB: 15–30 days	100/59600(0.18)	18/59600(0.19)	12/59600(0.13)	43/2748(0.36)	16/2748(0.4)	5/2748(0.28)
DB: 1–2 months	258/86467(0.33)	25/86467(0.18)	35/86467(0.26)	53/1947(0.62)	18/1947(0.63)	11/1947(0.86)
DB: 2–3 months	155/11628(1.47)	14/11628(0.74)	21/11628(1.15)	24/557(0.98)	4/557(0.49)	5/557(1.36)
DB > 3 months	176/7745(2.5)	8/7745(0.63)	31/7745(2.56)	20/312(1.46)	1/312(0.22)	5/312(2.44)
**Naturally-acquired or hybrid immunity[d] (ref.: unvaccinated without prior infection evidence)**						
Unvaccinated: NA	36/14292(0.28)	2/14292(0.09)	2/14292(0.09)	16/1332(0.27)	6/1332(0.31)	4/1332(0.46)
D1 or D2: NA	49/9084(0.59)	8/9084(0.54)	6/9084(0.42)	26/984(0.6)	6/984(0.42)	6/984(0.93)
DB: NA	76/16209(0.52)	9/16209(0.34)	8/16209(0.32)	14/512(0.62)	2/512(0.27)	1/512(0.3)

S2. Data description

Three National databases created to monitor the epidemic and the vaccination campaign were matched together.

SI-VIC, the information system for monitoring victims of attacks and exceptional health situations, provides, for people infected with SARS-CoV-2, the daily number of hospitalizations in general wards and ICU, and the number of inpatient deaths. The diagnosis of infection relies on RT-PCR testing or thoracic CT scanning. This reporting system, maintained by the ANS (*Agence du Numérique en Santé*), is exhaustive and covers all healthcare structures (public and private) over the French territory.

SI-DEP, the screening information system, provides the daily number of tests performed (RT-PCR, serology and antigenic tests) for SARS-CoV-2 and the results of these tests. This database, maintained by the AP-HP (*Assistance Publique - Hôpitaux de Paris*), is exhaustive for all tests performed on the French territory (but self-tests). Since mid-2020, PCR testing was available to the population without prescription and covered by national health insurance. As of January 2021, a molecular screening was performed on all RT-PCR positive samples: first to identify known variant strains (wild-type, alpha, beta, gamma); then, from June 2021, to identify some key mutations (E484K, E484Q, L452R). The presence or absence of symptoms in tested individuals should be systematically reported, but this information is missing for 20% of the RT-PCR tests performed in 2021.

VAC-SI, the Covid-19 vaccine information system, maintained by the CNAM, the French national Health Insurance (*Caisse Nationale d'Assurance Maladie*), provides the number of administrated vaccines and vaccinated persons on the French territory. This dataset covers nearly the entire French population (all those affiliated to the French Health Care System), whether vaccinated or not, and all individuals vaccinated in France. This database contains information on vaccination (dates of injection, vaccine brand name), and information on vaccine priority populations (presence of comorbidities, healthcare professionals or social workers, retirement homes residents).

To match these databases, a pseudonym (non-meaningful character string identifying each person) was generated from the concatenation and encryption of identifying information (surname, first name, sex and date of birth). The pseudonym (but not the identifying information) is present in all the databases transmitted to the Statistics office of the French Ministry for Solidarity and Health (DREES) for statistical use, which allows the matching of data on screening, hospitalization and vaccination at the individual level. However, matching imperfections may remain.

The deployment of these three databases was authorized by the French Data Protection Authority (*Commission Nationale Informatique et Libertés*). No consent of the patients is required, and the patients must be informed of their right to access, modify, rectify and delete any data concerning them. The French Ministry for Health is accountable to implement legal, technical and organizational measures to guarantee data protection.

Among persons aged 18 years or over hospitalized for Covid-19 (data source: SI-VIC), 75 % have a matched positive RT-PCR test, collected from fifteen days before admission to the end of their stay (source: SI-DEP) over our analysis period. Among persons aged 18 years or over hospitalized for Covid-19 (data source: SI-VIC), 70 % have a match in the VAC-SI register, which covers nearly all French residents and allows to recover the vaccination status. Among persons aged 18 years reporting symptoms in the last seven days before a RT-PCR test (data source: SI-DEP), 87 % have a match in the VAC-SI register, thus a known vaccination status.

In France, following the national surveillance strategy, part of positive RT-PCR samples are submitted to mutation screening in order to characterize the likely variant. A set of predefined mutations are targeted to identify the circulating variants with good confidence, and results are centralized into the SI-DEP databases. Over our study period, the following characteristics could be reported by laboratories for each analysed sample: detection of Spike mutation E484K, encoded as "A", detection of Spike mutation L452R encoded as "C", detection of at least one of Spike deletion 69-70; mutations K417N; N501Y ; S371L-S373P; Q493R encoded as "D", according to the following codes (0: not detected; 1: detected; 8: searched but inconclusive; 9: not searched). Code D was added to the operating system by on the 20th of December, but it took a few weeks before this modification was fully implemented. During the transition period, the absence of E484K and L452R (A0C0) was considered as a high suspicion for Omicron since none of these mutations is present on this strain. In this study, we consider individuals infected with SARS-CoV2 variant with A0C0 or D1 mutation profiles, as Omicron infections. Interpretable screened samples which did not meet criteria for Omicron were categorized as Delta cases.

People with co-morbidities are identified a priori by the CNAM, the French national Health Insurance based on recommendations of the HAS, the French Health Authority, to be prioritized for vaccination against COVID-19, because at high risk of a severe form of the disease or of death. Identification is carried out with health insurance data, based on long-term illnesses (ALD), or by targeting ICD codes. In particular, people with: diabetes, obesity, chronic renal failure, COPD and respiratory failure, arterial hypertension, heart failure, organ transplantation, cancer undergoing chemotherapy, are targeted. In addition to a priori identification, doctors could declare the presence of a comorbidity justifying a patient eligibility to vaccination.

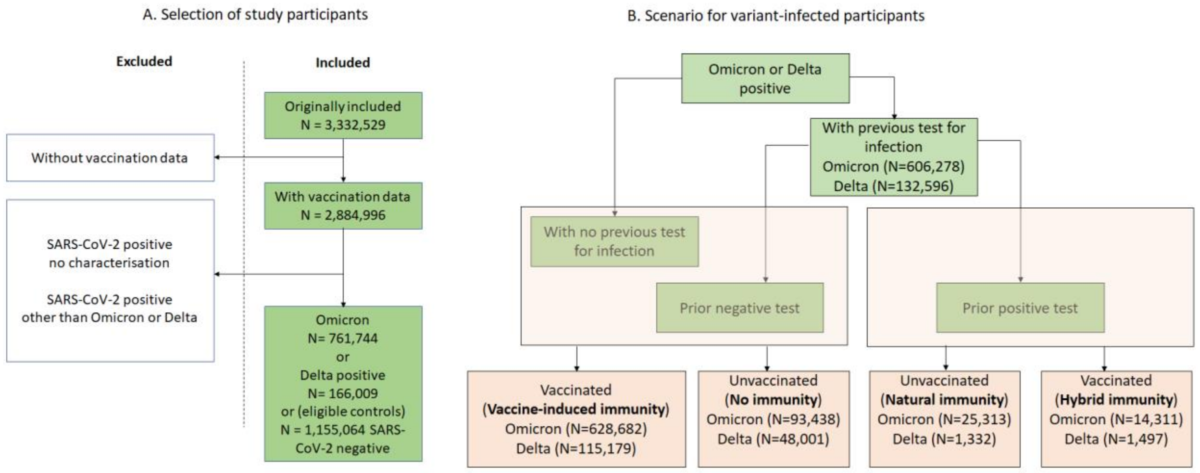

S 3. Supplementary results

Figure S-3.1 • Covid-19 vaccine effectiveness against Omicron (a) and Delta (b) symptomatic infections by age, according to the time elapsed since the injection of each vaccine dose, data collected from December 13, 2021 to January 31, 2022

(a) Omicron

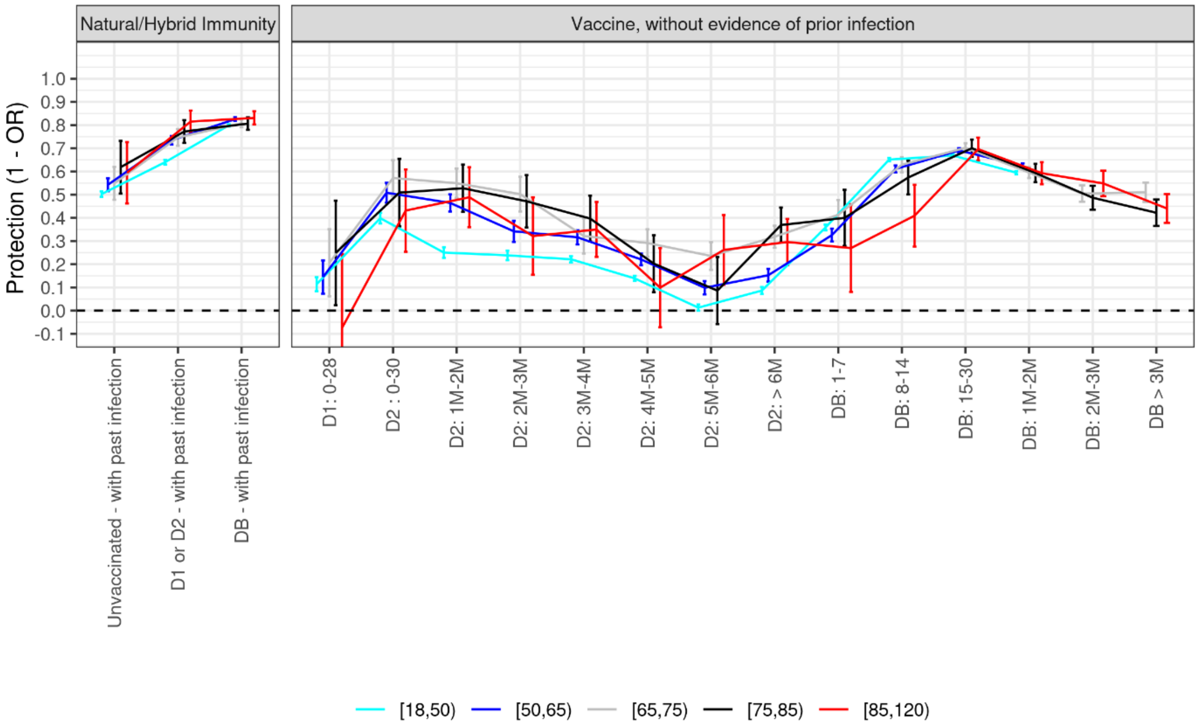

(b) Delta

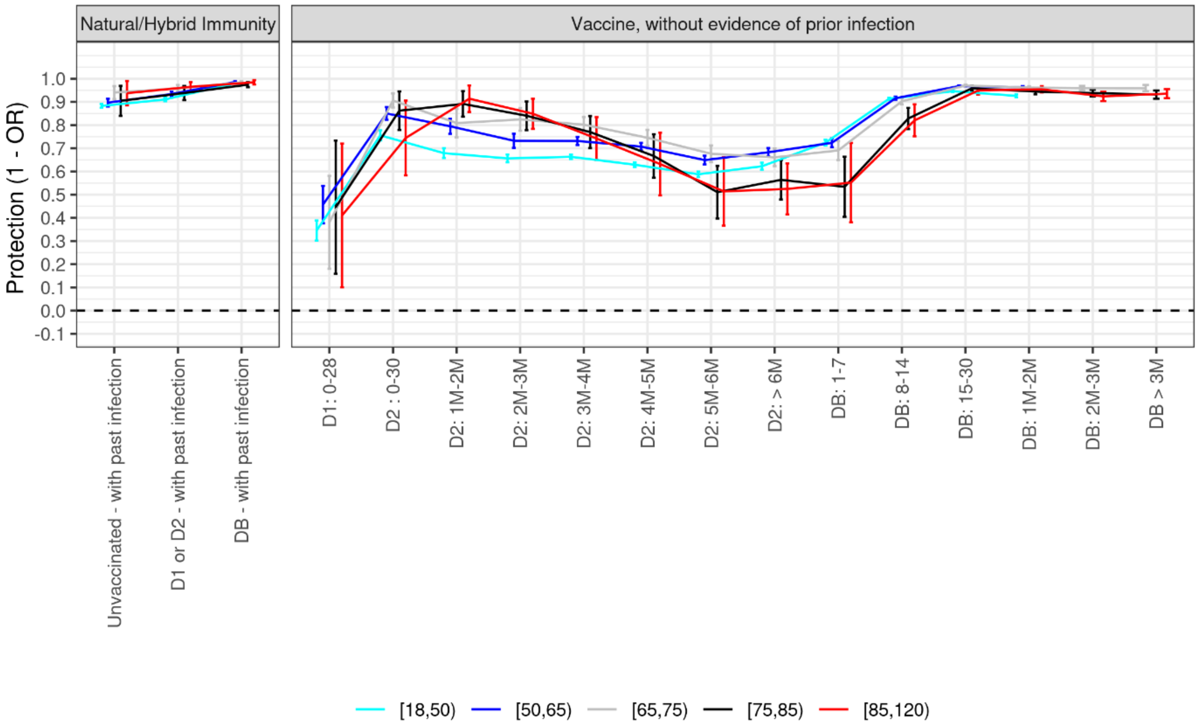

Figure S-3.2 • Variant-specific odd ratios of symptomatic infections and hazard ratios of hospitalizations after symptomatic infections among persons aged 18 years or over, according to liberal (baseline) or conservative definition of Omicron and Delta cases. Data from December 13, 2021 to January 31, 2022

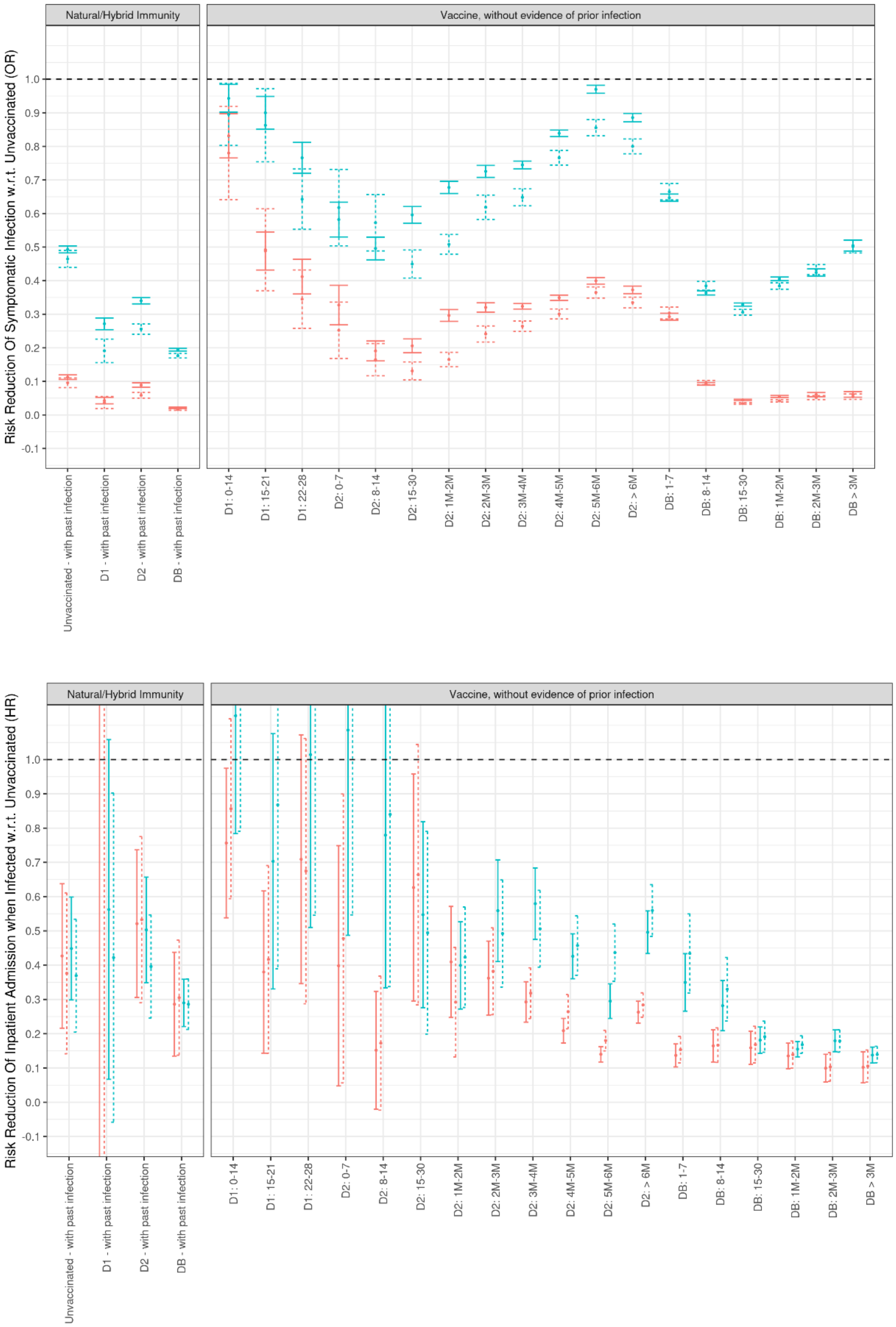

Protection against Omicron (blue) or Delta (red), according to this paper definition of cases (plain confidence intervals) or Auvigne et al. (2022) definition (dotted confidence intervals)
